# Oral Health and Dental Care Among Older Adults in China: What Are the Risk and Protective Factors?

**DOI:** 10.1093/geroni/igab046.111

**Published:** 2021-12-17

**Authors:** Weiyu Mao, Yaolin Pei, Huabin Luo

## Abstract

Oral health is a global public health concern. The four papers in this symposium capture various understudied risk and protective factors in oral health and dental care among older adults in China. The first paper examined the relationship between social isolation, loneliness, and tooth loss among Chinese older adults, using the Chinese Longitudinal Healthy Longevity Survey. The findings suggest that higher levels of social isolation, rather than loneliness, were associated with fewer remaining teeth and accelerated tooth loss over time. The second paper investigated urban-rural disparities in dental care utilization among Chinese adults aged 18 to 65 years old, using the 2019 New Era and Living Conditions in Megacities Survey. The findings demonstrate urban residents were more likely to visit dentists than rural residents. Besides socioeconomic status, health attitudes/behaviors, and oral health needs, health insurance coverage was considered an important enabling factor to promote dental care use in this population. The third paper examined the relationship between denture use and cognitive decline among Chinese older adults, using data from the Chinese Longitudinal Healthy Longevity Survey. The findings indicate that denture use is protective against cognitive decline over time in later life. The fourth paper examined the prevalence of self-reported orofacial pain symptoms and their correlates at the last year of life among Chinese older adults. Low socioeconomic status, smoking, chronic conditions, oral hygiene practice, and natural teeth condition were associated with such symptoms. This symposium offers valuable insights to improve oral health and dental care in older adults in China.

